# Supplementary material for: Comparison of Alere q whole blood viral load with DBS and plasma viral load in the classification of HIV virological failure
Source: PLoS One. 2020 May 29;15(5):e0232345. doi: 10.1371/journal.pone.0232345 (PMC7259604; doi:10.1371/journal.pone.0232345)
Supplement: S1 File — (DOCX) [file pone.0232345.s001.docx]

**Supporting information**

**Receiver operating characteristic (ROC) curve analysis:**

In light of the misclassification of Alere Q and DBS methodologies using the threshold of 1000 copies, we performed ROC curve analysis in order to understand the optimum threshold for classification of VF for each method. For the samples tested, 9249 copies/ml (3.97 log_10_) provided the best overall classification (85% correctly classified) for Alere q with a sensitivity of 82% and a specificity of 89%. For Abbott DBS, 1134 copies/ml (3.07 log_10_) provided the best overall classification (88% correctly classified) with a sensitivity of 90% and a specificity of 86%. For Roche FVE, 400 copies/ml (2.60 log_10_) was the optimal threshold with 82% correct classification, 92% sensitivity and 73% specificity.

**Fig.1 ROC curve analysis of Alere q and DBS assays using the threshold of 1000 copies/ml for VF**

**1a**

**1b**

**1c**

*ROC curve analysis for 1a) Alere q* shows area under ROC curve = 0.92 (95% CI 0.89-0.95), 1419 copies/ml (3.15 log_10_) and 44062 copies/ml (4.64 log_10_) provided 100% sensitivity and 100% specificity respectively. 1*b) Abbott DBS* area under ROC curve = 0.95 (0.93-0.97), 999 copies/ml (2.99 log_10_) and 5995 copies/ml (3.78 log_10_) provided 99% sensitivity and 100% specificity. *1c) Roche FVE* area under ROC curve = 0.90 (0.87-0.94), 400 copies/ml (2.60 log_10_) and 2134 copies/ml (3.33 log_10_) provided 92% sensitivity and 100% specificity.
