# Supplementary material for: Comparison of Alere q whole blood viral load with DBS and plasma viral load in the classification of HIV virological failure
Source: PLoS One. 2020 May 29;15(5):e0232345. doi: 10.1371/journal.pone.0232345 (PMC7259604; doi:10.1371/journal.pone.0232345)
Supplement: Attachment — Submitted filename: PONE response to reviewers 2nd revision.docx [file pone.0232345.s003.docx]

PONE-D-19-29100R1
Comparison of Alere q whole blood viral load with DBS and plasma viral load in the classification of HIV virological failure
PLOS ONE

Dear Dr Khan,

Thank you for submitting your manuscript to PLOS ONE. After careful consideration, we feel that it has merit but does not fully meet PLOS ONE’s publication criteria as it currently stands. Therefore, we invite you to submit a revised version of the manuscript that addresses the points raised during the review process.

Remaining issues to address include those of Reviewer 1 below. In particular, the study needs to include data from after 2015. While it is true that the data presented are from the earlier time, more studies have been published and the pooled results must be addressed to make this manuscript relevant to 2020.

Answer: Have included more recent references and studies.

The authors must also address the following issues. The samples included in the testing were all from HIV-infected individuals, although some were not detectable by the Abbott gold standard assay. The Abbott assay has both <40cp/mL detected and <40cp/mL not detected as result options, so it must be addressed if these two categories were combined as not detected or if they were split, putting the detected ones with the 40-1000cp/mL group. Overall, there must be a better description of how the data were compared. There is a description of the VF level, but it is unclear how that was applied to the data from samples with <1000cp/mL as the plasma VL. The authors must be very specific: how were the data from each of the assays treated to decide if they had the correct classification? In particular, Table 2 is confusing for this reason: for the <40 sample category, was it that 22% were correctly not detected by Alere (and the others were incorrectly detected) or that 22% were correctly detected by Alere (and the other were incorrectly not detected)? For the other groups, what was considered "correct classification", detected at any number or detected at a number in the same range (such as 1000-10,000)? Or were they correctly classified if they were above 1000cp/mL as the VF threshold? There are no figure legends to help describe the figures, and there are no differences in markers between the groups to see how the data in the table fit with the graphs. The authors must add more transparency to their results and calculations to make their data useful to the wider research community.

Answer: Categories changes based on reviewer comment (see revised manuscript).

Definition of correct classification of VF by Alere q and DBS methods according to plasma VL categories (table 2 ) = using the VF threshold of 1000 copies/ml:

when plasma VL < 1000 copies/ml (both target not detected and <40-1000copies/ml categories) then Alere q/DBS VL <1000 copies/ml (specificity in identifying absence of VF),

when plasma VL >= 1000 copies/ml (both 1000-10,000 and >10,000 copies/ml categories) then Alere q/DBS VL >1000 copies/ml (sensitivity in identifying VF). So Alere q correctly identified absence of VF in 22% of plasma VL in the target not detected category compared to 14% in the <40-1000copies/ml category.

Figure legends are in the manuscript as per submission requirements (page 8).

We would appreciate receiving your revised manuscript by Apr 10 2020 11:59PM. When you are ready to submit your revision, log on to [https://www.editorialmanager.com/pone/](https://protect-za.mimecast.com/s/rhC3C580KjtZ2qBkUzcUnO?domain=editorialmanager.com) and select the 'Submissions Needing Revision' folder to locate your manuscript file.

If you would like to make changes to your financial disclosure, please include your updated statement in your cover letter.

To enhance the reproducibility of your results, we recommend that if applicable you deposit your laboratory protocols in protocols.io, where a protocol can be assigned its own identifier (DOI) such that it can be cited independently in the future. For instructions see: [http://journals.plos.org/plosone/s/submission-guidelines#loc-laboratory-protocols](https://protect-za.mimecast.com/s/UobFC66VKkCrqQ5LT6K-3h?domain=journals.plos.org)

Please include the following items when submitting your revised manuscript:

- A rebuttal letter that responds to each point raised by the academic editor and reviewer(s). This letter should be uploaded as separate file and labeled 'Response to Reviewers'.
- A marked-up copy of your manuscript that highlights changes made to the original version. This file should be uploaded as separate file and labeled 'Revised Manuscript with Track Changes'.
- An unmarked version of your revised paper without tracked changes. This file should be uploaded as separate file and labeled 'Manuscript'.

Please note while forming your response, if your article is accepted, you may have the opportunity to make the peer review history publicly available. The record will include editor decision letters (with reviews) and your responses to reviewer comments. If eligible, we will contact you to opt in or out.

We look forward to receiving your revised manuscript.

Kind regards,

Julie AE Nelson, PhD
Academic Editor
PLOS ONE


[Note: HTML markup is below. Please do not edit.]

Reviewers' comments:

Reviewer's Responses to Questions

**Comments to the Author**

1. If the authors have adequately addressed your comments raised in a previous round of review and you feel that this manuscript is now acceptable for publication, you may indicate that here to bypass the “Comments to the Author” section, enter your conflict of interest statement in the “Confidential to Editor” section, and submit your "Accept" recommendation.

Reviewer #1: (No Response)

2. Is the manuscript technically sound, and do the data support the conclusions?

The manuscript must describe a technically sound piece of scientific research with data that supports the conclusions. Experiments must have been conducted rigorously, with appropriate controls, replication, and sample sizes. The conclusions must be drawn appropriately based on the data presented.

Reviewer #1: Yes

3. Has the statistical analysis been performed appropriately and rigorously?

Reviewer #1: Yes

4. Have the authors made all data underlying the findings in their manuscript fully available?

The [PLOS Data policy](https://protect-za.mimecast.com/s/rH-AC76JVlCAw3DGiBOAvm?domain=plosone.org) requires authors to make all data underlying the findings described in their manuscript fully available without restriction, with rare exception (please refer to the Data Availability Statement in the manuscript PDF file). The data should be provided as part of the manuscript or its supporting information, or deposited to a public repository. For example, in addition to summary statistics, the data points behind means, medians and variance measures should be available. If there are restrictions on publicly sharing data—e.g. participant privacy or use of data from a third party—those must be specified.

Reviewer #1: Yes

5. Is the manuscript presented in an intelligible fashion and written in standard English?

PLOS ONE does not copyedit accepted manuscripts, so the language in submitted articles must be clear, correct, and unambiguous. Any typographical or grammatical errors should be corrected at revision, so please note any specific errors here.

Reviewer #1: Yes

6. Review Comments to the Author

Please use the space provided to explain your answers to the questions above. You may also include additional comments for the author, including concerns about dual publication, research ethics, or publication ethics. (Please upload your review as an attachment if it exceeds 20,000 characters)

Reviewer #1: The authors have made great efforts to answer to the comments, and the article has improved considerably. I still have some minor comments however before the manuscript is deemed suitable for publication.

1. In the method section, the very first sentence is in contradiction with the following. Indeed, the authors state that the goals are to compare HIV viral load results obtained with Alere q POC with plasma HIV viral load results on the one hand, and with DBS viral load results on the other hand. However, two sentences later thy mention that secondary objectives are to compare DBS viral load results to plasma viral load results.
I guess the first sentence can be removed.

Answer: removed as per reviewer comment.

2. Line 73, when the authors say “the remaining paired CD4 sample was used for whole blood Alere q test…” I think that the terminlogy “CD4 sample was used” is not correct. Wouldn’t it be more accurate to say something like “the remainis of the blood sample for CD4 measurement”?

Answer: amended as per reviewer comment.

3. In current reference 14 (as cited in Table 1) , the limit of detection of the Abbott technique was found to be 839 copies/mL. This is also the threshold mentioned in the WHO pre-qualification document (1) This is also the technique specified in Taieb et al (PLOS one 2018) following the manufacturer’s information.

Answer: During the period of evaluation in 2014-2015, the Abbott RealTime HIV-1 DBS revised prototype protocol was under research use and not WHO pre-qualified. The limit of detection was reported at 1000 copies/ml (Shihai Huang SC, Brian Erickson, John Salituro, Chadwick Dunn, Livhuwani Nxumalo, Jeffrey Wuitschick, Jens Dhein. Improved Performance of the New Prototype Automated Abbott RealTime HIV-1 DBS Viral Load Assay: Potential Use in Expanded Viral Load Testing in Resource Limited Setting. ASLM2014) . Detectable results below the limit of detection were reported as <1000 copies/ml. WHO pre-qualification and CE marking of the one spot Abbott RealTime HIV-1 DBS protocol was done in 2016 in which the limit of detection was found to be 839 copies/ml (WHO prequalification of diagnostics programme public report 2016 and Tang N, Pahalawatta V, Frank A, Bagley Z, Viana R, Lampinen J, et al. HIV-1 viral load measurement in venous blood and fingerprick blood using Abbott RealTime HIV-1 DBS assay. Journal of Clinical Virology. 2017;92:56-61). Field evaluation by Taieb et al (PLOS one 2018) was done in 2017. Will remove reference Tang et al from Table 1 and discuss it under section 2.2 laboratory testing.

4. In the statistical method section, reference to ROC curve appears but no such result is presented. Consider removing the sentence referring to ROC curves.

Answer: Removed sentence referring to ROC curves.

5. In the results section, lines 140-144 are strangely placed. Consider moving them before the paragraph currently starting line 132.

Answer: Lines 140-144 are notes summarizing table 2. Can move it as per reviewer’s comment.

6. In the discussion, I do not understand why the authors specify that they stopped their literature search in June 2015. This does not make sense in 2020. Moreover, some articles evaluating the DBS techniques of interest have been published since. These articles should also be regarded especially since some articles fou,d similar results with the Roche technique (e.g. Taieb et al. OFID 2016).

Answer: Have included more recent studies and references as suggested. Pooled estimates of sensitivity and specificity have been changed to include more recent estimates reported in the World Health Organisation HIV molecular diagnostics toolkit to improve access to viral load testing and infant diagnosis 2019.

7. I still have issues with the Bland-Altman analysis. The figure should include the 95% confidence limits of the differences. I doubt the two horizontal grey lines correspond to these limits. The figures must be amended.

Answer: Have amended figures as advised.

(1) [https://www.who.int/diagnostics_laboratory/evaluations/pq-list/hiv-vrl/170830_amended_final_pr_0145_027_00.pdf](https://protect-za.mimecast.com/s/MVn-C8qYKmU6EnoNcM87e6?domain=who.int)

7. PLOS authors have the option to publish the peer review history of their article ([what does this mean?](https://protect-za.mimecast.com/s/k4h1C98XYntkDoG4UPAKiD?domain=journals.plos.org)). If published, this will include your full peer review and any attached files.


If you choose “no”, your identity will remain anonymous but your review may still be made public.


**Do you want your identity to be public for this peer review?** For information about this choice, including consent withdrawal, please see our [Privacy Policy](https://protect-za.mimecast.com/s/e1yFC0g3D4SGp61NU3qka-?domain=plos.org).

Reviewer #1: No


[NOTE: If reviewer comments were submitted as an attachment file, they will be attached to this email and accessible via the submission site. Please log into your account, locate the manuscript record, and check for the action link "View Attachments". If this link does not appear, there are no attachment files to be viewed.]


While revising your submission, please upload your figure files to the Preflight Analysis and Conversion Engine (PACE) digital diagnostic tool, [https://pacev2.apexcovantage.com/](https://protect-za.mimecast.com/s/x1sjCg5XMvfADVMEiZcMmF?domain=pacev2.apexcovantage.com). PACE helps ensure that figures meet PLOS requirements. To use PACE, you must first register as a user. Registration is free. Then, login and navigate to the UPLOAD tab, where you will find detailed instructions on how to use the tool. If you encounter any issues or have any questions when using PACE, please email us at [figures@plos.org](mailto:figures@plos.org). Please note that Supporting Information files do not need this step.
